# Bone and Mineral Metabolism Phenotypes in MEN1-Related and Sporadic Primary Hyperparathyroidism, before and after Parathyroidectomy

**DOI:** 10.3390/cells10081895

**Published:** 2021-07-26

**Authors:** Francesca Marini, Francesca Giusti, Federica Cioppi, Davide Maraghelli, Tiziana Cavalli, Francesco Tonelli, Maria Luisa Brandi

**Affiliations:** 1 Department of Experimental and Clinical Biomedical Sciences, University of Florence, 50139 Florence, Italy; francesca.marini@unifi.it (F.M.); francesca.giusti@unifi.it (F.G.); davide.maraghelli@unifi.it (D.M.); 2 F.I.R.M.O. Italian Foundation for the Research on Bone Diseases, 50141 Florence, Italy; francesco.tonelli@unifi.it; 3 SOD Bone and Mineral Metabolism Diseases, Azienda Ospedaliero Universitaria Careggi (AOUC), 50139 Florence, Italy; cioppif@aou-careggi.toscana.it; 4 General, Mini-Invasive and Emergency Surgery Unit, Department of Surgery and Orthopaedics, ASST “Carlo Poma”, 46100 Mantua, Italy; tiziana.cavalli@gmail.com

**Keywords:** multiple endocrine neoplasia type 1, primary hyperparathyroidism, parathyroidectomy, bone metabolism, bone mass

## Abstract

Primary hyperparathyroidism (PHPT) is the most common endocrinopathy in multiple endocrine neoplasia type 1 (MEN1). Persistent levels of increased parathyroid hormone (PTH) result in a higher incidence of osteopenia and osteoporosis compared to the general population. Surgical removal of hyper-functioning parathyroid tissue is the therapy of choice. This retrospective study evaluated the effect of parathyroidectomy (PTX) on bone metabolism and bone mass in two series of patients with MEN1 PHPT and sporadic PHPT (sPHPT) by comparing bone metabolism-related biochemical markers and bone mineral density (BMD) before and after surgery. Our data confirmed, in a higher number of cases than in previously published studies, the efficacy of PTX, not only to rapidly restore normal levels of PTH and calcium, but also to normalize biochemical parameters of bone resorption and bone formation, and to improve spine and femur bone mass, in both MEN1 PHPT and sPHPT. Evaluation of single-patient BMD changes after surgery indicates an individual variable bone mass improvement in a great majority of MEN1 PHPT patients. In MEN1 patients, PTX is strongly suggested in the presence of increased PTH and hypercalcemia to prevent/reduce the early-onset bone mass loss and grant, in young patients, the achievement of the bone mass peak; routine monitoring of bone metabolism and bone mass should start from adolescence. Therapy with anti-fracture drugs is indicated in MEN1 patients with BMD lower than the age-matched normal values.

## 1. Introduction

Primary hyperparathyroidism (PHPT) is a common endocrine disease characterized by persistent elevated levels of parathyroid hormone (PTH), leading to hypercalcemia. In a majority of cases (90–95%), PHPT manifests as a sporadic form (sPHPT), but it can also, though rarely, be part of familial isolated or syndromic diseases (5–10%) [1].

PHPT with multiple hyperplasia and/or adenoma of the parathyroid glands is the most common endocrinopathy of multiple endocrine neoplasia type 1 (MEN1), a rare inherited multi-organ tumor syndrome affecting the parathyroids, the anterior pituitary gland, and the neuroendocrine cells of the gastro-entero-pancreatic tract [2]. MEN1-related PHPT is estimated to account for about 2–4% of all cases of PHPT [3,4]. 

In MEN1, PHPT arises as the first clinical expression of the syndrome in about 90% of patients, with a mean age onset between 20 and 25 years, and symptomatic cases described already by the age of 8 [5], with an almost complete penetrance by the age of 50 [6]. In most cases, MEN1 PHPT can remain asymptomatic for a long period, but mild-moderate hypercalcemia typically starts during adolescence [7], and almost all MEN1 patients are expected to have hypercalcemia by the age of 50 [6].

Symptomatic PHPT manifests mainly with nephrolithiasis and/or bone involvement, with a higher susceptibility to both these clinical manifestations in MEN1 PHPT than in sPHPT [3,8,9]. High incidence (over 80%) of renal calculi was reported in MEN1 PHPT before the age of 30 years [8,10]. Bone demineralization in MEN1 PHPT is a frequent, early onset and progressive complication, which affects both trabecular and cortical bone [8]. By the age of 20–35 years, MEN1 women with PHPT showed a higher incidence of osteopenia and osteoporosis compared to the general population of the same age, with a consequent increased risk of fragility fracture [3,7,11]. A more severe degree of trabecular bone demineralization and lower values of both spine and femur bone mineral density (BMD) were reported in MEN1 PHPT compared to sPHPT patients, despite a significantly younger age, lower serum levels of PTH, and usually a milder hypercalcemia [3,9,12]. The earlier occurrence of MEN1 PHPT, about three decades before sPHPT, leading to persistent and prolonged increased levels of PTH, starting during late childhood, adolescence or early adulthood, may negatively affect the normal acquisition of bone mass peak, while in adults the increased bone resorption will be responsible for bone mass loss and increased bone fragility. Other associated MEN1-derived clinical conditions, such as hypogonadism, growth hormone deficiency, hypercortisolism, and/or nutrient malabsorption caused by surgical resection of the proximal tract of the small intestine, may worsen bone loss in MEN1 patients with PHPT [13]. Finally, a direct role of the *MEN1* gene mutations in altering the correct bone development and physiological regulation of bone remodeling cannot be excluded [13].

The surgical excision of hyper-functioning parathyroid tissue (parathyroidectomy; PTX) is indicated to prevent clinical complications due to prolonged elevated levels of PTH and calcium, such as bone loss, nephrolithiasis and hypercalcemia-induced neuromuscular, heart and brain alterations. Parathyroid surgery is the only decisive therapy for PHPT, and it should be considered in all patients, being recommended in symptomatic cases. PTX is also indicated for asymptomatic PHPT, both in sporadic and inherited syndromic forms, in presence of a worsening hypercalcemia or with serum total calcium of 1.0 mg/dL above the upper limit of the normal value [14]. Currently, successful PTX is the elective and most employed therapy, both for MEN1 PHPT and sPHPT [15].

In sPHPT, generally caused by single parathyroid adenoma, the indicated surgery procedure is adenomectomy only of the affected glands. Conversely, multiglandular removal by subtotal PTX (SPTX; removal of 3–3.5 parathyroids) or total PTX (TPTX; removal of all the four glands with or without heterotopic implant of part of the not-tumor parathyroid gland in the non-dominant forearm) are the two most frequently employed surgeries in MEN1 [16]. Given the genetic nature of MEN1 and the multiple affected parathyroids in this syndrome, PHPT persistence or recurrence after PTX is observed in 20–60% of patients, compared to only a 4% recurrence rate reported in sPHPT [17].

In this retrospective study, we compared the bone and mineral metabolism in two series of MEN1 PHPT and sPHPT patients and analyzed the impact of PTX on bone metabolism phenotypes and bone mass in these two forms of PHPT. The results promote attentive evaluation of the bone phenotype in MEN1 PHPT patients at any age, as well as early surgery.

## 2. Material and Methods

### 2.1. Patients

Clinical data of MEN1 patients with PHPT, followed up at the Regional Referral Center for Inherited Endocrine Tumors of the Tuscany Region in Florence, were retrospectively extracted from the “Florentine MEN1 database” [18], as part of the “Italian MEN1 Database” [19], which was approved by the Review Board of the “Area Vasta Centro, Regione Toscana” at the “Azienda Ospedaliera-Universitaria Careggi” (Rif. CEAVC OSS 16.234). Patients had signed an informal consent form before their data were retrieved from their medical records and retrospectively and prospectively included in the Italian MEN1 Database. Surgical interventions were performed in different hospitals, and data about parathyroid surgeries and post-operative follow-up were retrieved by medical records and collected within the MEN1 database at the time of patients’ insertion and follow-up controls.

Before inclusion in the MEN1 database, a diagnosis of MEN1 was performed according to one of the following criteria: (1) endocrine tumors in at least two of the main affected organs; (2) one endocrine tumor in one of the main affected organs and one first-grade relative with MEN1; or (3) identification of a germinal inactivating mutation of the *MEN1* gene.

A total of 133 MEN1 patients (46 men and 87 women) were affected by PHPT at the time of the present study and were included in the analyses; 105 underwent at least one PTX during the retrospective observation period of this study.

Forty-seven sPHPT patients (3 men and 44 women), defined as having an idiopathic non-syndromic hyperparathyroidism, without any family history of PHPT, and having in their medical records a DXA evaluation performed before PTX or any medical therapy for PHPT, were selected for the study.

Medical data of both MEN1 PHPT and sPHPT patients were retrospectively retrieved, respectively, from the MEN1 database or individual medical records, and analyzed anonymously.

For the present study, we selectively collected data about age, gender, familial and clinical history, onset age of PHPT, surgical interventions of the parathyroid glands (including age at surgery, type of PTX, eventual persistence and/or recurrences and re-intervention(s)), medical therapy for PHPT, and presence of nephrolithiasis. All MEN1-related clinical manifestations and therapies were collected as well.

### 2.2. Biochemical Measurements

We collected data about serum and urinary biomarkers of bone and mineral metabolism (i.e., serum: PTH, total calcium, calcium ion, phosphorus, bone alkaline phosphatase, 25-hydroxy-vitamin D; urine: calcium, phosphorus, deoxypyridinoline). For patients who did not undergo parathyroid surgery, we collected the last biochemical measurements available in the medical records. For operated patients, we retrieved both pre-operative and post-operative biochemical data, considering the last measurement performed before PTX and the first one performed after surgery, respectively. The referral normal values for these biochemical parameters are indicated in Table 1, Table 2 and Table 3.

### 2.3. Bone Densitometry Measurements

Available data on BMD values and T-score, measured by Dual-energy X-ray Absorptiometry (DXA) (Delphi QDR Series, HOLOGIC, Marlborough, MA, USA) at lumbar spine (L1–L4), femoral neck and total femur, were collected. For patients who did not undergo parathyroid surgery, we considered, for this study, the last performed DXA. In operated patients, the available pre-operative and post-operative DXA data were collected. If more than one DXA evaluation was performed before the PTX, we considered, for this study, the last one performed before surgery. If data from a second DXA evaluation after PTX were available for patients, they were collected and compared with pre-operative data as well. Patients were classified as osteoporotic if at least one of the three measured bone sites presented a T-score ≤ −2.5, osteopenia with at least one T-score higher than −2.5 but lower than −1.0, and normal BMD if all the three measured bone sites had a T-score ≥ −1.0 [20].

Available data on fragility fractures were retrieved as well, both for MEN1 PHPT and sPHPT.

### 2.4. Statistical Analysis

Biochemical and instrumental parameters were all analyzed by descriptive statistics; data are presented as nominal categories, mean ± standard deviation (SD) or percentages. Statistical comparisons of bone and mineral parameters among MEN1 PHPT and sPHPT patients, as well as between data collected before and after PTX, were performed by using the Student’s *t*-test for parametric values, assuming a *p* value less than 0.05 as an indicator of statistical significance (over 95%) and a *p* value less than 0.01 as an indicator of high statistical significance (over 99%).

## 3. Results 

### 3.1. Clinical Data

Out of the 133 MEN1 PHPT cases, 87 were women (65.4%) and 46 men (34.6%), with a female:male ratio of 1.89:1. PHPT was diagnosed at a mean age of 34.1 ± 13.5 years (range 11–66 years).

Twenty-eight MEN1 PHPT patients (21.1%) did not undergo PTX at the time of their last follow-up at the Referral Center (mean age 41.1 ± 18.0 years, range 13–77 years); all of them showed no instrumental evidence of PHPT at their last neck control (i.e., no enlarged parathyroids were detected at their last ultrasonography and/or Tc99m-sestamibi scintigraphy scan of the neck). Eighteen (64.3%) had a normocalcemic PHPT, while ten (35.7%) manifested slightly increased serum calcium (range 10.2–11.2 mg/dL).

There were 105 (78.9%) MEN1 PHPT patients who underwent at least one PTX in the study period 1991–2020, with a mean age at surgery of 36.6 ± 14.3 years (range 12–66 years). The mean gap between the diagnosis of PHPT and the first parathyroid surgery was 2.2 ± 5.2 years. The main indication for PTX was hypercalcemia, with or without recurrent nephrolithiasis and/or severe osteoporosis. Operated MEN1 patients included 73 women (69.5%) and 32 men (30.5%), with a female:male ratio of 2.28:1. There was no significant difference between the female:male ratio of PHPT incidence and female:male ratio of PTX, indicating that there was not a difference between genders in undergoing the parathyroid surgery. There were 39 MEN1 patients who underwent a TPTX (37.1%), 41 a SPTX (39.0%), and 25 a partial PTX (PPTX; 23.8%). Out of the 105 MEN1 PHPT surgeries, we reported, in the post-operative follow-up, 21 cases of recurrence (20.0%), 12 cases of persistence (11.4%), and 13 cases of permanent hypoparathyroidism (12.4%). Fifteen MEN1 PHPT patients (14.3%) underwent a second PTX; eleven of them (73.3%) were re-operated for a PHPT recurrence and four (26.7%) to correct a persistent PHPT. After the second surgery, only one patient presented a further PHPT recurrence that was treated with cinacalcet, while six cases (40.0%) manifested post-surgical permanent hypoparathyroidism.

The 47 sPHPT patients manifested the first clinical sign of PHPT at a mean age of 59.4 ± 13.1 years (range 39–83 years). They included 44 women (93.6%) and 3 men (6.4%), with a female:male ratio of 14.67:1.

Eighteen sPHPT patients (38.3%) did not undergo PTX at the time of this study. Nine of them had a normocalcemic PHPT, while nine manifested a slightly increased serum calcium (range 10.2–11.4 mg/dL).

A total of 29 of the 47 sPHPT patients (61.7%) underwent at least one PTX (mean age at surgery 55.9 ± 12.4 years). All the 29 surgeries were single gland PPTXs, with the selective ablation of only the affected parathyroid. Operated sPHPT patients presented a female:male ratio of 28.0:1, 28 women (96.6%) and one man (3.4%). Out of the 29 operated sPHPT patients, 4 showed a post-operative persistence of PHPT (13.8%), 2 manifested recurrences of PHPT (6.9%), and 4 presented a post-surgical transient hypoparathyroidism (13.8%). One woman (3.4%) required three PTXs for correction of PHPT recurrences.

### 3.2. Pre-Operative Bone and Mineral Metabolism

Comparison of serum and urinary bone and mineral metabolism-related parameters, measured before PTX or in PHPT patients who did not undergo PTX, between MEN1 PHPT and sPHPT patients, is reported in Table 1 as mean ± SD.

MEN1 PHPT patients showed a significantly lower average serum level of phosphate, in association with a significantly higher urinary excretion of phosphate, compared to sPHPT.

MEN1 PHPT patients presented levels of bone alkaline phosphatase (BALP) over the normal range, and significantly higher than sPHPT individuals, in whom average serum levels of this enzyme are within the normal value.

MEN1 PHPT patients on average had vitamin D insufficiency (less than 30.0 ng/mL), while sPHPT patients on average had normal levels of vitamin D. All MEN1 PHPT and sPHPT patients with marked vitamin D insufficiency (less than 20.0 ng/mL) or deficiency (less than 10.0 ng/mL) were supplemented with this hormone, according to guidelines, to help control PTH oversecretion [14].

### 3.3. Effects of PTX on Bone and Mineral Metabolism

Table 2 and Table 3 report comparisons between mean levels of serum and urinary bone and mineral metabolism-related parameters, measured before and after PTX, in 105 MEN1 PHPTs and 29 sPHPTs, respectively, as well as mean percentage change of each parameter after surgery.

After PTX, in both MEN1 PHPT patients (Table 2) and sPHPT patients (Table 3), there were highly significant reductions of serum levels of PTH, calcium ion, total calcium, BALP (all these parameters moving back within their normal values), and urinary deoxypyridinoline, associated with a significant increase in serum phosphate and a significant reduction of urinary excretion of calcium.

### 3.4. Pre-Operative Bone Mass

Parameters from a pre-operative DXA analysis were available only for 53 MEN1 PHPT patients (25 non-operated and 28 operated). For 13 additional MEN1 PHPT patients, indication of the presence of pre-operative osteopenia or osteoporosis and age at diagnosis were reported in their medical records, in absence of DXA numeric parameters.

Out of these total 66 MEN1 PHPT patients:

- 27 cases (40.9%) presented osteoporosis (mean age at measurement 52.0 ± 14.7 years, range 23–79 years);
- 29 cases (43.9%) presented osteopenia (mean age at measurement 39.1 ± 13.7 years, range 20–76 years);
- 10 cases (15.2%) had normal BMD values (mean age at measurement 28.9 ± 10.5 years, range 13–43 years).

MEN1 PHPT patients with a normal BMD showed a significantly lower mean age compared to both MEN1 patients with osteoporosis (*t*-test *p*-value = 0.00006) and MEN1 patients with osteopenia (*t*-test *p* value = 0.039). MEN1 PHPT patients with osteopenia presented a significantly lower mean age of onset than those with osteoporosis (*t*-test *p*-value = 0.00013).

Out of the 47 sPHPT patients:

- 31 cases (66.0%) presented osteoporosis (mean age at measurement 64.4 ± 9.2 years, range 53–86 years);
- 13 cases (27.6%) presented osteopenia (mean age at measurement 56.4 ± 12.0 years, range 39–73 years);
- 3 cases (6.4%) had normal BMD values (mean age at measurement 63.5 ± 12.0 years, range 55–72 years).

No significant differences of mean age were found among the three groups of sPHPT patients.

Comparisons of BMD values and T-scores, measured before PTX or in PHPT patients who did not undergo PTX, between MEN1 PHPT and sPHPT patients, are reported in Table 4 as mean ± SD.

Despite their significantly younger age, MEN1 PHPT patients showed aggregated mean values of BMD and T-scores, at all the measured bone sites, comparable with those of sPHPT patients.

### 3.5. Effects of PTX on Bone Mass

Parameters from a pre-operative and a first post-operative DXA analysis were available for 28 of the 105 operated MEN1 PHPT patients (26.7%); data from a second post-operative DXA evaluation were available for 16 of these patients. As a consequence of the fact that this is a retrospective study in which we retrieved, from medical records, only the pre- and post-operative DXA data that were available, timing of the post-operative DXA examination was extremely variable among our patients (mean 28.3 + 24.5 months; range 8–94 months). Data of a post-operative DXA performed within the first 12 months after surgery were available for only 8 MEN1 PHPT patients, and they were analyzed as well, separately.

Table 5 reports comparisons of mean values of BMD and T-scores, measured before and after PTX, in MEN1 PHPTs.

Comparison of aggregate DXA data in MEN1 PHPT, before and after surgery, showed that PTX induced an improvement of bone mass, at all the evaluated bone sites, both in the total group of analyzed patients and in the 8 patients with a post-operative DXA performed within 12 months after surgery. BMD mean percentage changes showed no significant differences between the two groups of patients at lumbar spine (*p* = 0.905), femoral neck (*p* = 0.649) and total femur (*p* = 0.752).

Analysis of individual BMD variations, before and after surgery, showed a high variability between single patients, as indicated by elevated SDs of the mean percentage changes between pre-operative and post-operative values. All patients but one showed, singularly, a post-operative improvement of lumbar spine BMD. Three (3/28; 10.7%) and four patients (4/28; 14.3%) showed a post-operative worsening of bone mass at total femur and femoral neck, respectively.

One woman showed a post-operative worsening of BMD, at all the 3 bone sites, in 2 different DXA evaluations performed 9 and 22 months after PTX. A BMD improvement was found between the first and second post-operative DXA at all the three bone sites (+5.0% at lumbar spine, +7.7% at femoral neck, and +10.8% at total femur). She developed a pituitary adenoma secreting ACTH, causing Cushing’s syndrome, at the age of 37 years, and was diagnosed with PHPT two years later. The last pre-operative DXA was performed 16 months before PTX at the age of 40. She underwent PPTX (ablation of the superior right gland) at the age of 41. Post-operative follow-up showed a two year-persistence of hyperparathyroidism (secondary to a deficit of vitamin D and calcium), perhaps responsible for the lack of BMD regain after surgery.

Parameters from pre-operative and post-operative DXA analyses were available for all the 29 operated sPHPT patients. Table 6 reports comparisons of mean values of BMD and T-scores, measured before and after PTX, in sPHPTs.

As for MEN1 PHPT, comparison of aggregate DXA data in sPHPT, before and after surgery, showed that PTX induced an improvement of bone mass at all the evaluated bone sites, without reaching a statistical significance.

## 4. Discussion

Many patients affected by PHPT, either in syndromic or sporadic forms, lack classical signs and symptoms traditionally associated with excessive PTH secretion and/or hypercalcemia (asymptomatic PHPT). In some cases, the absence of symptoms can be an indication to postpone PTX, mostly in normocalcemic younger patients, to delay until later in life the risk of post-operative hypoparathyroidism [14]. The latest guidelines (2013) for the management of asymptomatic PHPT recommend PTX also for asymptomatic cases in the presence of specific biochemical and clinical conditions, indicating a risk for bone health and/or development of nephrolithiasis or nephrocalcinosis [14]. A total of 25 of the 105 operated MEN1 PHPT patients and 11 of the 29 operated sPHPT patients resulted asymptomatic at the time of their last control before surgery; a serum level of total calcium of 1.0 mg/dL higher than the upper limit of normal value and/or a worsening hypercalcemia were the indications for parathyroid surgery for all of them.

MEN1 PHPT and sPHPT differ in clinical features, with the main discriminant between these two forms of PHPT being the age of onset. Our study confirmed the earlier occurrence of MEN1 PHPT than sPHPT, with a mean age gap of about 15 years between the two groups of patients. In addition, the male:female ratio was confirmed to be different in MEN1 PHPT vs. sPHPT, with a significantly higher prevalence of affected women in sPHPT (93.6% of cases in our series) compared to MEN1 PHPT (65.4% of cases). However, the very high female:male ratio of 14.67:1 of our sPHPT series (compared to the 3:1 ratio commonly reported for sPHPT) could be due to the fact that, for this study, we selected only sPHPT patients who had a DXA evaluation in their medical records, a medical exam that is more commonly performed in females than males. 

In our series, MEN1 PHPT showed a lower PTH mean value and a significantly lower serum level of phosphate than sPHPT, as reported by a previous study [3]. Conversely to that study [3], we found a prevalence of nephrolithiasis in MEN1 PHPT, higher than in sPHPT but about half as low as that reported in MEN1 PHPT patients in previously studies [8,10].

As opposed to other studies [3,9], no significant differences in BMD values and T-score were reported between our MEN1 PHPT and sPHPT patients at all the analyzed bone sites. However, according to results from Wang et al. [12], our MEN1 PHPT patients had a significantly lower Z-score at lumbar spine and femoral neck than sPHPT (data not shown). Since Z-score represents the standard deviation of the attenuation of X-ray passage through bone tissue compared to that of the healthy population of the same age, our results confirmed that MEN1 PHPT is responsible for reduction of bone mass, both at cortical and trabecular compartments, at any age, leading to a demineralized bone compared with the average bone density of healthy people of the same age and gender, and increasing the risk for early fragility fractures, significantly more than sPHPT.

In our study, MEN1 PHPT and sPHPT patients, either before PTX or not undergoing parathyroid surgery, exhibited elevated levels of PTH, total and ionized calcium, and deoxypyridinoline, indicating an increased bone turnover. Untreated MEN1 PHPT was also characterized by pre-operative increased serum levels of BALP, indicating in these patients the presence of a higher activity of osteoblasts. Conversely, in untreated sPHPT, BALP levels were within the normal range. The significantly younger age of MEN1 PHPT patients could explain higher levels of BALP, but a direct role of menin deficiency cannot be excluded. Indeed, menin has been demonstrated to induce the early commitment of mesenchymal stem cells to osteoblast lineage, while it inhibits the later differentiation of committed osteoblasts. Menin directly interacts with JunD, a component of the activator protein-1 (AP-1), a transcription factor complex, which plays an important role in skeletal modeling and remodeling, and this interaction leads to a repression of JunD-activated transcription. JunD expression gradually increases during osteoblast differentiation and induces expression of the Runx2 differentiation marker, the collagen type 1 and osteocalcin proteins of the extracellular bone matrix, and the pro-mineralization enzyme alkaline phosphatase (ALP) [21]. Reduction of the expression/activity of wild type menin in *MEN1* mutated patients may result in an up-regulation of JunD and an increase of ALP expression and BALP serum levels, and also be directly responsible for a not-correctly controlled late stage of the osteoblast differentiation process, which may result in the alteration of osteoblast–osteoclast coupled activity in MEN1 patients, with respect to sPHPT.

MEN1 PHPT patients on average had vitamin D insufficiency (less than 30.0 ng/mL), while sPHPT on average had normal levels of vitamin D. MEN1 PHPT usually has a long normocalcemic and asymptomatic period, during which the elevated levels of PTH can “consume” the 25-hydroxy-vitamin D by converting it into 1,25-dihydroxy-vitamin D. This mechanism could explain why our MEN1 PHPT patients showed a pre-operative insufficiency of vitamin D, with respect to the sporadic counterpart, despite the fact that all MEN1 PHPT and sPHPT patients with vitamin D less than 20.0 ng/mL received a supplementation of this hormone.

Comparison of bone and mineral metabolism-related biochemical parameters, measured before and after PTX, in our two series of PHPT patients confirmed that parathyroid surgery is efficient in restoring the normal PTH secretion and normalizing total calcium and calcium ion levels, as reported by previous studies [22,23,24,25], in association, in our patients, with a significant reduction in urinary excretion of calcium, in both MEN1 PHPT and sPHPT cases. A study by Coutinho et al. [23] also showed a post-operative decrease in urinary calcium, but without reaching statistical significance. PTX also normalized serum levels of BALP in our MEN1 PHPT, suggesting a restoration of normal osteoblast activity. Urinary deoxypyridinoline, an indicator of bone resorption, decreased after surgery in MEN1 PHPT and sPHPT, even if it remained slightly higher than normal range in both the groups.

Bone health in MEN1 syndrome is still poorly investigated, as demonstrated by the fact that two of the most important and well-known national MEN1 databases, the GENEM in France [26] and the Dutch MEN1 Study Group (DMSG) in The Netherlands [27], are both missing the collection and analysis of bone mass parameters. A study from the French and Belgian GENEM study group’s MEN1 database [28] evaluated surgical trends and results of parathyroid surgery in 256 MEN1 patients with PHPT; data regarding bone status and bone mass were totally missing before and after the surgery, except for the biochemical markers of mineral metabolism directly related to parathyroid function, such as PTH and serum calcium. Recently, a multicentric study by Saponaro et al. [29] collected clinical, biochemical, and instrumental data on 604 Italian patients with sPHPT and familial, non-syndromic and syndromic PHPT, including 23 cases of MEN1 PHPT. They analyzed, as aggregated pre-operative clinical data, the presence of osteoporosis, the occurrence of clinical fractures and some biochemical markers of mineral metabolism, together with other clinical characteristics, in the whole group, in the group of symptomatic PHPT, and in the group of asymptomatic PHPT, as possible indications for PTX. However, no single data regarding only the MEN1 PHPT group, or comparing sPHPT with the inherited forms of parathyroid disease, were reported in the study.

To date, in the three papers that included information on BMD variation before and after PTX in MEN1 PHPT patients, the numbers of analyzed cases were respectively 5, 14 and 16 [7,17,25]. Our retrospective study compared pre- and post-operative DXA bone health parameters in 28 MEN1 PHPT and 29 sPHPT patients. The fact that a pre-operative DXA evaluation was missing in the medical records of 77 (73.3%) of the 105 MEN1 PHPT operated patients, mostly those operated before 2014 and/or who came to the attention of our Referral Center after the PTX had already been performed, confirmed that for a long time the assessment of bone status has been considered a marginal aspect to be evaluated in MEN1 patients with PHPT by clinicians. This aspect is confirmed also by the fact that only 8 operated MEN1 PHPT patients had a post-operative DXA performed within 12 months after parathyroid surgery, as it should be for a correct evaluation of the effect of PTX on bone mass recovery.

All the three previously published studies [7,17,25] reported a post-operative recovery of BMD at lumbar spine and femoral neck in MEN1 patients with PHPT. In a review that analyzed the post-surgical follow-up of MEN1 PHPT, Coutinho et al. [15] concluded that there is a short-term marked improvement of spinal and femoral BMD in a majority of patients, usually after a successful TPTX with autologous transplant. Our study confirmed, in a relatively large number of MEN1 cases, a general post-operative improvement of BMD at lumbar spine, femoral neck and total femur, in both the syndromic and sporadic parathyroid diseases—improvement which, in MEN1 PHPT patients, appeared to progressively increase with time, as assessed at the second DXA measurement performed after surgery. Bone mass recovery in MEN1 PHPT patients appeared more pronounced at lumbar spine, a site of preponderant trabecular bone, than at femoral neck, a skeletal site predominantly of cortical bone. Comparison of pre-operative and post-operative DXA parameters in our PHPT MEN1 cases indicated that the degree of amelioration of bone-mass loss after PTX is largely variable among single patients, and it is influenced by the time elapsing between the PHPT diagnosis and surgical intervention. A prompt ablation of parathyroid adenoma at the first sign of alteration in bone and mineral metabolism, in the presence of a hypercalcemic PHPT, and/or when PHPT develops at a very young age (affecting the normal bone modeling and the achievement of bone mass pick), is mandatory to prevent/reduce the early onset bone mass loss. Indeed, in our series, patients with pre-surgery advanced osteoporosis showed a more limited recovery of bone mass and, especially in younger individuals, a not-achievable restoration of age-appropriate bone mineralization, suggesting that when bone mass is already compromised, the beneficial effect of PTX on BMD is limited, and the risk of an established osteoporosis and fragility fracture increases.

The presence of MEN1-related endocrine comorbidities, other than PHPT, and the administered therapies, could further negatively influence bone phenotype, mostly if these diseases develop during adolescence and young adulthood, and/or they could negatively influence the bone mass regain after PTX.

Among our MEN1 PHPT patients was a male young adult (25 years) manifesting hypercalcemic PHPT from the age of 16 and a pituitary macroadenoma secreting high levels of prolactin from the age of 12, with a very severe osteoporosis at lumbar spine (T- and Z-scores both −4.4) and osteoporosis at total femur and femoral neck. This peculiar MEN1 clinical phenotype, starting in very early adolescence, could have severely altered normal skeleton modeling and may explain the severe bone affection in this young patient, including a fragility fracture of the radius occurring at the age of 25. PTX was performed at the age of 25, after 24 months of pharmacological treatment with cinacalcet that failed to control PTH over-secretion. After 20 months from PTX, only a slight gain of spine and femur BMD was found, enforcing the hypothesis that he did not achieve the correct bone-mass peak during his skeleton development, and this may have reduced the beneficial efficacy of PTX on BMD.

We also reported the case of a 41-year-old woman who underwent a PPTX of a single gland, in which both of the two DXA measurements performed, respectively, 9 and 22 months after surgery, showed a worsening of the Z-score and T-score at lumbar spine, femoral neck and total femur, compared to the pre-operative evaluations. Only a small improvement of BMD was recorded between the first and second post-operative DXA. She had a pituitary adenoma secreting ACTH, which had developed two years before diagnosis of PHPT, causing Cushing’s syndrome, which could explain the lack of BMD regain after surgery and the worsening of bone mass [30].

Extensive epidemiological and clinical studies of bone phenotypes, bone metabolism and BMD values, before and after PTX, in MEN1 PHPT are important to improve medical knowledge in this specific area of the syndrome, and to favor the design of appropriate individual prevention and management of bone complications, reducing and/or avoiding the impact of early onset fragility fractures. These studies should be designed as prospective epidemiological investigations, consisting of the targeted collection of pre- and post-operative bone and mineral metabolism-related data at specific and standardized months before and after PTX. In addition, the integration of databases of MEN1 patients with bone and mineral metabolism-related clinical data will allow an increase in the number of cases analyzed, enabling an empowerment of analysis, reducing any underestimation of differences, and also granting the possibility to study stratified subgroups of patients.

From a clinical point of view, the screening of bone metabolism and osteoporosis risk should be routinely included in the diagnostic plan of MEN1 patients with PHPT, together with analysis for oncological monitoring. Data from this study suggest a therapeutic intervention for MEN1 PHPT, even if still asymptomatic, by surgery and/or calcimimetics [31], to control PTH secretion early in the evolution of PHPT, preferably associated with the administration of anti-fracture drugs when BMD levels are severely lower than age- and gender-matched normal values. 

Last but not least, a post-operative long-term follow-up of parathyroid function, bone and mineral metabolism, and bone mass status must be included in MEN1 routine management, with the final goal of avoiding fragility fractures during the lifetimes of these patients.

## Figures and Tables

**Table 1:** Comparison of serum and urinary bone and mineral metabolism-related parameters, measured before PTX or in PHPT patients who did not undergo PTX, between MEN1 PHPT and sPHPT.

Parameter ^1^	MEN1 PHPT (*n* = 133)	sPHPT (*n* = 47)	*p* Value MEN1 PHPT vs. sPHPT
Age at measurement (years)	38.9 ± 16.1	64.3 ± 14.1	**<0.01**
Parathyroid hormone (1.3–7.6 pmol/L)	17.2 ** ± 17.2	19.0 ** ± 18.0	Not significant
Serum calcium ion (4.3–5.3 mg/dL)	5.67 ** ± 0.43	5.55 ** ± 0.35	Not significant
Total serum calcium (8.5–10.1 mg/dL)	10.5 ** ± 1.5	10.3 ** ± 0.9	Not significant
Serum phosphate (2.5–4.9 mg/dL)	2.5 ± 0.6	2.7 ± 0.3	**<0.01**
25-hydroxy-vitamin D (30–100 ng/mL)	21.7 * ± 11.6	33.7 ± 11.2	**<0.01**
Bone alkaline phosphatase (Men: 7–20 µg/L; pre-menopausal women: 4–14.3 µg/L; post-menopausal women: 6–22.5 µg/L)	24.1 ** ± 14.1	17.2 ± 7.4	**<0.05**
Urinary calcium (42–353 mg/24 h)	328.7 ± 155.4	269.5 ± 123.7	Not significant
Urinary phosphate (400–1300 mg/24 h)	780.3 ± 286.9	641.4 ± 210.3	**<0.05**
Urinary deoxypyridinoline (3.0–5.4 mnol/mmol creatinine)	7.5 ** ± 3.7	7.3 ** ± 3.1	Not significant
Nephrolithiasis	63/133 (47.4%)	15/47 (31.9%)	Not significant

^1^ = normal values are indicated within brackets. * indicates a parameter value less than the normal range; ** indicate a parameter value higher than the normal range. The statistical significance (*p* value) is indicated in bold.

**Table 2:** Comparison of serum and urinary bone and mineral metabolism-related parameters, measured before and after PTX, in MEN1 PHPT patients.

Parameter ^1^	Pre-Surgery MEN1 PHPT (*n* = 105)	Post-Surgery MEN1 PHPT (*n* = 105)	*p* Value Pre-Surgery vs. Post-Surgery	Mean Percentage Change between Pre-Surgery and Post-Surgery Values
Age at measurement (years)	34.4 ± 14.0	36.4 ± 13.8	Non applicable	Non applicable
Gap between measurement and surgery (months)	5.3 ± 5.2	12.41 ± 12.8	Non applicable	Non applicable
Parathyroid hormone (1.3–7.6 pmol/L)	20.0 ** ± 19.7	5.2 ± 3.3	**<0.01**	−64.6 ± 29.7%
Serum calcium ion (4.3–5.3 mg/dL)	5.84 ** ± 0.41	4.88 ± 0.50	**<0.01**	−16.0 ± 10.7%
Total serum calcium (8.5–10.1 mg/dL)	10.7 ** ± 0.9	9.0 ± 0.8	**<0.01**	−15.0 ± 11.1%
Serum phosphate (2.5–4.9 mg/dL)	2.3 * ± 0.5	3.0 ± 0.5	**<0.01**	+37.9 ± 33.4%
25-hydroxy-vitamin D (30–100 ng/mL)	17.4 * ± 8.0	30.0 ± 15.3	**<0.01**	+97.8 ± 117.0%
Bone alkaline phosphatase (Men: 7–20 µg/L; pre-menopausal women: 4–14.3 µg/L; post-menopausal women: 6–22.5 µg/L)	29.3 ** ± 17.1	18.5 ± 12.2	**<0.05**	−25.5 ± 65.1%
Urinary calcium (42–353 mg/24 h)	353.4 ** ± 124.1	210.4 ± 89.3	**<0.01**	−24.9 ± 74.1%
Urinary phosphate (400–1300 mg/24 h)	755.3 ± 205.1	768.4 ± 262.2	Not significant	+11.6 ± 51.9%
Urinary deoxypyridinoline (3.0–5.4 mnol/mmol creatinine)	8.9 ** ± 3.1	5.9 ** ± 1.5	**<0.01**	−29.8 ± 19.4%

^1^ = normal values are indicated within brackets. * indicates a parameter value less than the normal range; ** indicate a parameter value higher than the normal range. The statistical significance (*p* value) is indicated in bold.

**Table 3:** Comparison of serum and urinary bone and mineral metabolism-related parameters, measured before and after PTX, in sPHPT patients.

Parameter ^1^	Pre-Surgery sPHPT (*n* = 29)	Post-Surgery sPHPT (*n* = 29)	*p* Value Pre-Surgery vs. Post-Surgery	Mean Percentage Change between Pre-Surgery and Post-Surgery Values
Age at measurement (years)	58.2 ± 13.7	59.2 ± 13.1	Non applicable	Non applicable
Gap between measurement and surgery (months)	5.4 ± 3.6	7.2 ± 10.3	Non applicable	Non applicable
Parathyroid hormone (1.3–7.6 pmol/L)	18.8 ** ± 17.2	6.5 ± 3.2	**<0.01**	−55.7 ± 26.7%
Serum calcium ion (4.3–5.3 mg/dL)	5.72 ** ± 0.18	4.76 ± 0.46	**<0.01**	−16.6 ± 9.3%
Total serum calcium (8.5–10.1 mg/dL)	10.5 ** ± 0.7	9.4 ± 0.7	**<0.01**	−10.7 ± 7.7%
Serum phosphate (2.5–4.9 mg/dL)	2.7 ± 0.4	3.3 ± 0.5	**<0.01**	+28.1 ± 28.5%
25-hydroxy-vitamin D (30–100 ng/mL)	33.3 ± 8.4	37.0 ± 11.8	Not significant	+17.9 ± 48.1%
Bone alkaline phosphatase (Men: 7–20 µg/L; pre-menopausal women: 4–14.3 µg/L; post-menopausal women: 6–22.5 µg/L)	24.1 ** ± 8.3	12.3 ± 2.9	**<0.01**	−43.5 ± 27.0%
Urinary calcium (42–353 mg/24 h)	322.7 ± 129.6	213.1 ± 141.7	**<0.05**	−28.9 ± 40.7%
Urinary phosphate (400–1300 mg/24 h)	678.3 ± 224.3	397.0 ± 108.1	Not significant	+38.9 ± 15.3%
Urinary deoxypyridinoline (3.0–5.4 mnol/mmol creatinine)	9.4 ** ± 2.2	6.9 ** ± 1.0	**<0.05**	−22.4 ± 28.2%

^1^ = normal values are indicated within brackets. ** indicate a parameter value higher than the normal range. The statistical significance (*p* value) is indicated in bold.

**Table 4:** Comparison of DXA parameters, measured before PTX or in PHPT patients who did not undergo PTX, between MEN1 PHPT and sPHPT.

Parameter	MEN1 PHPT (*n* = 53)	sPHPT (*n* = 47)	*p* Value MEN1 PHPT vs. sPHPT
Age at measurement (years)	42.6 ± 16.1	63.2 ± 11.3	**<0.01**
Lumbar spine BMD (g/cm^2^)	0.884 ± 0.154	0.855 ± 0.133	Not significant
Lumbar spine T-score	−1.7 ± 1.4	−2.1 ± 1.2	Not significant
Femoral neck BMD (g/cm^2^)	0.704 ± 0.120	0.702 ± 0.150	Not significant
Femoral neck T-score	−1.7 ± 0.9	−1.9 ± 1.2	Not significant
Total femur BMD (g/cm^2^)	0.843 ± 0.177	0.816 ± 0.141	Not significant
Total femur T-score	−1.3 ± 1.0	−1.5 ± 0.9	Not significant

The statistical significance (*p* value) is indicated in bold.

**Table 5:** Comparison of DXA parameters, measured before and after PTX, in MEN1 PHPT.

Parameter	Pre-Surgery MEN1 PHPT ^1^ (*n* = 28)	Post-Surgery MEN1 PHPT ^1^ (*n* = 28)	*p* Value Pre-Surgery vs. Post-Surgery ^1^	Mean Percentage Change between Pre-Surgery and Post-Surgery BMD ^1^	Pre-Surgery MEN1 PHPT ^2^ (*n* = 16)	Post-Surgery MEN1 PHPT ^2^ (*n* = 16)	*p* Value Pre-Surgery vs. Post-Surgery ^2^	Mean Percentage Change between Pre-Surgery and Post-Surgery BMD ^2^	Pre-Surgery MEN1 PHPT ^3^ (*n* = 8)	Post-Surgery MEN1 PHPT ^3^ (*n* = 8)	*p* Value Pre-Surgery vs. Post-Surgery ^2^	Mean Percentage Change between Pre-Surgery and Post-Surgery BMD ^2^
Age at measurement (years)	42.3 ± 15.1	45.3 ± 14.2	Non applicable	Non applicable	42.4 ± 15.6	47.6 ± 14.4	Non applicable	Non applicable	49.3 ± 14.5	50.5 ± 14.3	Non applicable	Non applicable
Gap between measurement and surgery (months)	7.5 ± 12.1 (before)	28.3 ± 24.5 (after)	Non applicable	Non applicable	9.3 ± 14.1 (before)	55.4 ± 32.5 (after)	Non applicable	Non applicable	7.1 ± 5.7 (before)	9.8 ± 1.4 (after)	Non applicable	Non applicable
Lumbar spine BMD (g/cm^2^)	0.818 ± 0.157	0.879 ± 0.164	Not significant	+7.7 ± 6.0%	0.776 ± 0.138	0.862 ± 0.145	Not significant	+11.5 ± 8.4%	0.773 ± 0.131	0.832 ± 0.135	Not significant	+8.0 ± 9.4%
Lumbar spine T-score	−2.3 ± 1.3	−1.7 ± 1.4	Not significant	Non applicable	−2.6 ± 1.2	−1.8 ± 1.3	Not significant	Non applicable	−2.6 ± 1.2	−2.1 ± 1.2	Not significant	Non applicable
Femoral neck BMD (g/cm^2^)	0.673 ± 0.114	0.697 ± 0.128	Not significant	+4.2 ± 9.2%	0.656 ± 0.110	0.689 ± 0.130	Not significant	+6.1 ± 5.3%	0.633 ± 0.081	0.665 ± 0.125	Not significant	+6.1 ± 11.3%
Femoral neck T-score	−1.9 ± 0.9	−1.6 ± 1.0	Not significant	Non applicable	−2.1 ± 0.9	−1.6 ± 1.0	Not significant	Non applicable	−2.1 ± 0.8	−1.8 ± 1.1	Not significant	Non applicable
Total femur BMD (g/cm^2^)	0.801 ± 0.161	0.841 ± 0.170	Not significant	+5.4 ± 10.0%	0.752 ± 0.121	0.810 ± 0.121	Not significant	+8.9 ± 5.1%	0.753 ± 0.062	0.809 ± 0.153	Not significant	+7.0 ± 14.3%
Total femur T-score	−1.6 ± 0.9	−1.2 ± 1.0	Not significant	Non applicable	−1.8 ± 0.9	−1.2 ± 0.9	Not significant	Non applicable	−1.7 ± 0.5	−1.3 ± 1.1	Not significant	Non applicable

^1^ = data are referred to the first DXA after PTX. ^2^ = data are referred to the second DXA after PTX. ^3^ = data are referred only to operated MEN1 PHPT patients with a first post-operative DXA analysis performed within 12 months after surgery.

**Table 6:** Comparison of DXA parameters, measured before and after PTX, in sPHPT.

Parameter	Pre-Surgery sPHPT (*n* = 29)	Post-Surgery sPHPT (*n* = 29)	*p* Value Pre-Surgery vs. Post-Surgery	Mean Percentage Change between Pre-Surgery and Post-Surgery BMD
Age at measurement (years)	67.7 ± 5.9	71.0 ± 4.6	Non applicable	Non applicable
Gap between measurement and surgery (months)	26.3 ± 30.3 (before)	11.7 ± 11.0 (after)	Non applicable	Non applicable
Lumbar spine BMD (g/cm^2^)	0.765 ± 0.109	0.784 ± 0.085	Not significant	+2.6 ± 2.9%
Lumbar spine T-score	−2.8 ± 0.4	−2.4 ± 0.6	Not significant	Non applicable
Femoral neck BMD (g/cm^2^)	0.717 ± 0.205	0.728 ± 0.225	Not significant	+2.8 ± 11.6%
Femoral neck T-score	−2.6 ± 1.2	−2.1 ± 0.7	Not significant	Non applicable
Total femur BMD (g/cm^2^)	0.711 ± 0.089	0.724 ± 0.054	Not significant	+3.5 ± 1.1%
Total femur T-score	−2.3 ± 0.6	−1.7 ± 1.0	Not significant	Non applicable

## Data Availability

The dataset used and analyzed during the current study is available from the corresponding author on reasonable request.

## References

[1] Walker M.D., Silverberg S.J. (2018). Primary hyperparathyroidism. Nat. Rev. Endocrinol..

[2] Kamilaris C.D.C., Stratakis C.A. (2019). Multiple Endocrine Neoplasia Type 1 (MEN1): An Update and the Significance of Early Genetic and Clinical Diagnosis. Front. Endocrinol..

[3] Eller-Vainicher C., Chiodini I., Battista C., Viti R., Mascia M.L., Massironi S., Peracchi M., D’Agruma L., Minisola S., Corbetta S. (2009). Sporadic and MEN1-related primary hyperparathyroidism: Differences in clinical expression and severity. J. Bone Miner. Res..

[4] Giusti F., Tonelli F., Brandi M.L. (2012). Primary hyperparathyroidism in multiple endocrine neoplasia type 1: When to perform surgery?. Clinics.

[5] Goudet P., Dalac A., Le Bras M., Cardot-Bauters C., Niccoli P., Levy-Bohbot N., du Boullay H., Bertagna X., Ruszniewski P., Borson-Chazot F. (2015). MEN1 disease occurring before 21 years old: A 160-patient cohort studyfrom the groupe d’etude des tumeurs endocrines. J. Clin. Endocrinol. Metab..

[6] Thakker R.V., Newey P.J., Walls G.V., Bilezikian J., Dralle H., Ebeling P.R., Melmed S., Sakurai A., Tonelli F., Brandi M.L. (2012). Clinical practice guidelines for multiple endocrine neoplasia type 1 (MEN1). J. Clin. Endocrinol. Metab..

[7] Burgess J.R., David R., Greenaway T.M., Parameswaran V., Shepherd J.J. (1999). Osteoporosis in multiple endocrine neoplasia type 1: Severity, clinical significance, relationship to primary hyperparathyroidism, and response to parathyroidectomy. Arch. Surg..

[8] Lourenço D.M., Coutinho F.L., Toledo R.A., Gonçalves T.D., Montenegro F.L.M., Toledo S.P.A. (2012). Biochemical, bone and renal patterns in hyperparathyroidism associated with multiple endocrine neoplasia type 1. Clinics.

[9] Lourenço D.M., Coutinho F.L., Toledo R.A., Montenegro F.L., Correia-Deur J.E., Toledo S.P.A. (2010). Early-onset, progressive, frequent, extensive, and severe bone mineral and renal complications in multiple endocrine neoplasia type 1-associated primary hyperparathyroidism. J. Bone Miner. Res..

[10] Christopoulos C., Antoniou N., Thempeyioti A., Calender A., Economopoulos P. (2005). Familial multiple endocrine neoplasia type I: The urologist is first on the scene. BJU Int..

[11] Kann P.H., Bartsch D., Langer P., Waldmann J., Hadji P., Pfützner A., Klüsener J. (2012). Peripheral bone mineral density in correlation to disease-related predisposing conditions in patients with multiple endocrine neoplasia type 1. J. Endocrinol. Invest..

[12] Wang W., Nie M., Jiang Y., Li M., Meng X., Xing X., Wang O., Xia W. (2020). Impaired Geometry, Volumetric Density, and Microstructure of Cortical and Trabecular Bone Assessed by HR-pQCT in Both Sporadic and MEN1-related Primary Hyperparathyroidism. Osteoporos. Int..

[13] Maraghelli D., Giusti F., Marini F., Brandi M.L. (2020). Bone tissue and mineral metabolism in hereditary endocrine tumors: Clinical manifestations and genetic bases. Orphanet J. Rare Dis..

[14] Bilezikian J.P., Brandi M.L., Eastell R., Silverberg S.J., Udelsman R., Marcocci C., Potts J.T. (2014). Guidelines for the management of asymptomatic primary hyperparathyroidism: Summary statement from the Fourth International Workshop. J. Clin. Endocrinol. Metab..

[15] Coutinho F.L., Lourenço D.M., Toledo R.A., Montenegro F.L., Toledo S.P.A. (2012). Post-surgical follow-up of primary hyperparathyroidism associated with multiple endocrine neoplasia type 1. Clinics.

[16] Choi H.R., Choi S.H., Choi S.M., Kim J.K., Lee C.R., Kang S.W., Lee J., Jeong J.J., Nam K.H., Chung W.Y. (2020). Benefit of diverse surgical approach on short-term outcomes of MEN1-related hyperparathyroidism. Sci. Rep..

[17] Silva A.M., Vodopivec D., Christakis I., Lyons G., Wei Q., Waguespack S.G., Petak S.M., Grubbs E., Lee J.E., Perrier N. (2017). Operative intervention for primary hyperparathyroidism offers greater bone recovery in patients with sporadic disease than in those with multiple endocrine neoplasia type 1-related hyperparathyroidism. Surgery.

[18] Marini F., Giusti F., Brandi M.L. (2018). Multiple endocrine neoplasia type 1: Extensive analysis of a large database of Florentine patients. Orphanet J. Rare Dis..

[19] Giusti F., Cianferotti L., Boaretto F., Cetani F., Cioppi F., Colao A., Davì M.V., Faggiano A., Fanciulli G., Ferolla P. (2017). Multiple endocrine neoplasia syndrome type 1: Institution, management, and data analysis of a nationwide multicenter patient database. Endocrine.

[20] National Institutes of Health, Osteoporosis and Related Bone Diseases National Resource Center Bone Mass Measurement: What the Numbers Mean. https://www.bones.nih.gov/sites/bones/files/pdfs/bonemassmeasure-508.pdf.

[21] Naito J., Kaji H., Sowa H., Hendy G.N., Sugimoto T., Chihara K. (2005). Menin suppresses osteoblast differentiation by antagonizing the AP-1 factor, JunD. J. Biol. Chem..

[22] Lamas C., Navarro E., Casterás A., Portillo P., Alcázar V., Calatayud M., Álvarez-Escolá C., Sastre J., Boix E., Forga L. (2019). MEN1-associated Primary Hyperparathyroidism in the Spanish Registry: Clinical Characterictics and Surgical Outcomes. Endocr. Connect..

[23] Coutinho F.L., Lourenço D.M., Toledo R.A., Montenegro F.L., Correia-Deur J.E., Toledo S.P.A. (2010). Bone mineral density analysis in patients with primary hyperparathyroidism associated with multiple endocrine neoplasia type 1 after total parathyroidectomy. Clin. Endocrinol..

[24] Silverberg S.J., Gartenberg F., Jacobs T.P., Shane E., Siris E., Staron R.B., McMahon D.J., Bilezikian J.P. (1995). Increased bone mineral density after parathyroidectomy in primary hyperparathyroidism. J. Clin. Endocrinol. Metab..

[25] Guo C.Y., Thomas W.E., al-Dehaimi A.W., Assiri A.M., Eastell R. (1996). Longitudinal changes in bone mineral density and bone turnover in postmenopausal women with primary hyperparathyroidism. J. Clin. Endocrinol. Metab..

[26] Chanson P., Cadiot G., Murat A. (1997). Management of patients and subjects at risk for multiple endocrine neoplasia type 1: MEN 1. GENEM 1. Groupe d’Etude des Néoplasies Endocriniennes Multiples de type 1. Horm. Res..

[27] Goudet P., Cougard P., Vergès B., Murat A., Carnaille B., Calender A., Faivre J., Proye C. (2001). Hyperparathyroidism in multiple endocrine neoplasia type I: Surgical trends and results of a 256-patient series from Groupe D’etude des Néoplasies Endocriniennes Multiples Study Group. World J. Surg..

[28] Pieterman C.R.C., van Hulsteijn L.T., den Heijer M., van der Luijt R.B., Bonenkamp J.J., Hermus A.R.M.M., Rinkes I.H.M.B., Vriens M.R., Valk G.D., DutchMEN1 Study Group (2012). Primary hyperparathyroidism in MEN1 patients: A cohort study with longterm follow-up on preferred surgical procedure and the relation with genotype. Ann. Surg..

[29] Saponaro F., Cetani F., Repaci A., Pagotto U., Cipriani C., Pepe J., Minisola S., Cipri C., Vescini F., Scillitani A. (2018). Clinical presentation and management of patients with primary hyperparathyroidism in Italy. J. Endocrinol. Invest..

[30] Rahaman S.H., Jyotsna V.P., Kandasamy D., Shreenivas V., Gupta N., Tandon N. (2018). Bone Health in Patients with Cushing’s Syndrome. Indian J. Endocrinol. Metab..

[31] Giusti F., Cianferotti L., Gronchi G., Cioppi F., Masi L., Faggiano A., Colao A., Ferolla P., Brandi M.L. (2016). Cinacalcet therapy in patients affected by primary hyperparathyroidism associated to Multiple Endocrine Neoplasia Syndrome type 1. Endocrine.

